# Supplementary material for: Temporal Range Dynamics of the Lataste’s Viper (Vipera latastei Boscá, 1878) in Doñana (Spain): Insights into Anthropogenically Driven Factors
Source: Animals (Basel). 2024 Oct 19;14(20):3025. doi: 10.3390/ani14203025 (PMC11504652; doi:10.3390/ani14203025)
Supplement: Supplementary file 1 [file animals-14-03025-s001.zip › animals-3221046-supplementary.pdf]

Supplementary material

Temporal range dynamics of Lataste´s viper (*Vipera latastei* Boscá, 1878) in Doñana (Spain): insights into anthropogenic-driven factors

Rafael Carmona-González[1], Francisco Carro[1], Juan Pablo González de la Vega[2], Fernando Martínez-Freiría[3,4]

1. Estación Biológica de Doñana (EBD-CSIC). Avda. Américo Vespucio, 45 41092, Seville, Spain; 2. Avda. Andalucía, 70. 4ºD. 21007, Huelva, Spain; 3. CIBIO, Centro de Investigação em Biodiversidade e Recursos Genéticos, InBIO Laboratório Associado, Universidade do Porto, Vairão, Portugal; 4. BIOPOLIS Program in Genomics, Biodiversity and Land Planning, CIBIO, Vairão, Portugal.

Correspondence: Rafael Carmona-González: rafael.carmona@ebd.csic.es; Fernando Martínez-Freiría: fmartinez-freiria@cibio.up.pt

TABLES

Table S1 - Bioclimatic variables derived in this study, depicting code, meaning (and units) and ranges of variation for the historical and contemporary periods.

CODE	Meaning (and units)	Historical	Contemporary
**BIO 01**	Annual Mean Temperature (ºC)	29.079-29.192	29.118-29.226
**BIO 02**	Mean Diurnal Range (Mean of monthly (max temp-min temp)	1.126-0.56	1.142-0.561
**BIO 03**	Isothermality (BIO2/BIO7) x100 (coefficient of variation)	31.701-40.047	30.429-39.418
**BIO 04**	Temperature Seasonality (Standard Deviation x 100)	43.514-59.024	46.709-63.663
**BIO 05**	Max Temperature of Warmest Month (ºC)	30.036-30.697	30.131-30.805
**BIO 06**	Min Temperature of Coldest Month (ºC)	27.866-28.269	27.886-28.287
**BIO 07**	Temperature Annual Range (BIO5-BIO6) (ºC)	1.767-2.814	1.844-2.902
**BIO 08**	Mean Temperature of Wettest Quarter (ºC)	28.515-28.705	28.803-28.967
**BIO 09**	Mean Temperature of Driest Quarter (ºC)	29.628-29.896	29.71-30
**BIO 10**	Mean Temperature of Warmest Quarter (ºC)	29.67-29.918	29.726-30
**BIO 11**	Mean Temperature of Coldest Quarter (ºC)	28.415-28.603	28.42-28.608
**BIO 12**	Annual precipitation (mm)	513.321-647.229	534.315-688.163
**BIO 13**	Precipitation of Wettest Month (mm)	94.80-119.27	84.749-104.784
**BIO 14**	Precipitation of Driest Month (mm)	3.751-1.274	0.881-0.498
**BIO 15**	Precipitation Seasonality (Coefficient of Variation)	72.966-77.987	66.263-70.08
**BIO 16**	Precipitation of Wettest Quarter (mm)	263.25-327.586	241.967-299.384
**BIO 17**	Precipitation of Driest Quarter (mm)	12.21-20.875	9.2-16.371
**BIO 18**	Precipitation of Warmest Quarter (mm)	23.58-35.559	9.845-37.987
**BIO 19**	Precipitation of Coldest Quarter (mm)	220.366-270.874	179.329-242.685

Table S2 - NDVI variables derived in this study, depicting names and ranges of variation for the historical and contemporary periods.

NDVI variable	Historical	Contemporary
Spring mean	-0.499 - 0.568	-0.503 - 0.637
Spring Standard Deviation	0.02 - 0.304	0.028 - 0.279
Summer Mean	-0.431 - 0.652	-0.429 - 0.726
Summer Standard Deviation	0.023 - 0.199	0.012 - 0.226
Autumn Mean	-0.528 - 0.594	-0.518 - 0.592
Autumn Standard Deviation	0.03 - 0.238	0.026 - 0.271

Table S3 - Performance evaluation of the historical model based on features and regularization parameters.

fc	rm	tune.args	auc.train	cbi.train	auc.diff.avg	auc.diff.sd	auc.val.avg	auc.val.sd	cbi.val.avg	cbi.val.sd	or.10p.avg	or.10p.sd	or.mtp.avg	or.mtp.sd	AICc	delta.AICc	w.AIC	ncoef	
1	L	1	fc.L_rm.1	0.86269662	0.907	0.06922389	0.05324601	0.8679828	0.06892624	0.76975	0.08638817	0.103125	0.14732864	0.046875	0.09375	675.213531	0	0.14671836	5
2	Q	1	fc.Q_rm.1	0.8648181	0.952	0.06834808	0.05254017	0.86947628	0.06780467	0.754	0.13454615	0.103125	0.14732864	0.046875	0.09375	679.322556	4.10902504	0.01880273	7
3	P	1	fc.P_rm.1	0.86715738	0.957	0.06552568	0.05037848	0.87169005	0.06514743	0.76725	0.083934	0.0875	0.11814539	0.046875	0.09375	681.843902	6.63037117	0.00532988	8
4	LQ	1	fc.LQ_rm.1	0.86524562	0.95	0.0672052	0.05401505	0.86847186	0.06741982	0.75825	0.10722989	0.103125	0.14732864	0.046875	0.09375	688.27959	13.066059	0.00021342	10
5	LQP	1	fc.LQP_rm.1	0.86672985	0.962	0.06687233	0.05391244	0.87034401	0.06705807	0.7745	0.0624313	0.103125	0.14732864	0.046875	0.09375	681.599083	6.38555244	0.00602392	8
6	H	1	fc.H_rm.1	0.89691458	0.871	0.04991911	0.0558067	0.87252093	0.05435936	0.78625	0.09012722	0.153125	0.17688715	0.0625	0.125	750.76226	75.5487287	5.77E-18	26
7	LQH	1	fc.LQH_rm.1	0.89780995	0.937	0.05076862	0.05390725	0.87867548	0.0542138	0.769	0.07615773	0.128125	0.15491093	0.046875	0.09375	705.274494	30.0609627	4.35E-08	20
8	L	2	fc.L_rm.2	0.86276115	0.904	0.0692626	0.05273025	0.86837526	0.06870252	0.7575	0.08349651	0.103125	0.14732864	0.046875	0.09375	675.378138	0.16460715	0.13512648	5
9	Q	2	fc.Q_rm.2	0.86460031	0.956	0.06799423	0.05276733	0.86927937	0.06752195	0.7365	0.14995444	0.103125	0.14732864	0.046875	0.09375	677.01669	1.80315869	0.0595571	6
10	P	2	fc.P_rm.2	0.8674155	0.93	0.06396116	0.04826523	0.87218673	0.06298321	0.75075	0.10459565	0.103125	0.14732864	0.046875	0.09375	675.376756	0.16322459	0.13521992	5
11	LQ	2	fc.LQ_rm.2	0.86456804	0.95	0.06696555	0.0539948	0.86837229	0.06719032	0.77475	0.11798128	0.103125	0.14732864	0.046875	0.09375	685.711164	10.4976333	0.00077082	9
12	LQP	2	fc.LQP_rm.2	0.86660079	0.964	0.06553387	0.05327741	0.8701804	0.0662455	0.7845	0.07265214	0.103125	0.14732864	0.046875	0.09375	679.974763	4.76123171	0.01357051	7
13	H	2	fc.H_rm.2	0.88216907	0.795	0.05797458	0.05665819	0.87351162	0.06164049	0.842	0.03397058	0.13958333	0.16292679	0.0625	0.125	690.299559	15.0860279	7.77E-05	14
14	LQH	2	fc.LQH_rm.2	0.88182222	0.88	0.05536261	0.05479255	0.87406693	0.05930456	0.80775	0.03063087	0.11875	0.17721809	0.046875	0.09375	698.780853	23.5673217	1.12E-06	16
15	L	3	fc.L_rm.3	0.86303541	0.929	0.06898258	0.05241064	0.86857677	0.06828847	0.777	0.09764903	0.103125	0.14732864	0.046875	0.09375	675.640192	0.4266605	0.11853219	5
16	Q	3	fc.Q_rm.3	0.86447931	0.947	0.06730342	0.0526202	0.86911755	0.06677362	0.76975	0.07893193	0.103125	0.14732864	0.046875	0.09375	677.491435	2.27790385	0.04697253	6
17	P	3	fc.P_rm.3	0.86818182	0.954	0.061129	0.0451206	0.87214232	0.05975079	0.73425	0.12610677	0.103125	0.14732864	0.046875	0.09375	675.379484	0.16595286	0.13503559	4
18	LQ	3	fc.LQ_rm.3	0.86458417	0.939	0.06653415	0.05346806	0.86851312	0.06661933	0.7875	0.06996428	0.103125	0.14732864	0.046875	0.09375	686.215735	11.0022037	0.00059894	9
19	LQP	3	fc.LQP_rm.3	0.86689925	0.961	0.0646127	0.0523614	0.87004904	0.06558703	0.79	0.06833252	0.103125	0.14732864	0.046875	0.09375	681.452194	6.23866312	0.006483	7
20	H	3	fc.H_rm.3	0.87683714	0.787	0.06100654	0.05392222	0.87055958	0.06309318	0.81575	0.07926485	0.13958333	0.16292679	0.0625	0.125	683.643847	8.4303157	0.00216703	10
21	LQH	3	fc.LQH_rm.3	0.87127128	0.928	0.0612031	0.05443129	0.86899605	0.0640046	0.78825	0.05518076	0.103125	0.14732864	0.046875	0.09375	703.345598	28.132067	1.14E-07	15
22	L	4	fc.L_rm.4	0.86300315	0.904	0.06844892	0.05236949	0.86845617	0.06774545	0.808	0.07514874	0.103125	0.14732864	0.046875	0.09375	675.99328	0.77974849	0.09934917	5
23	Q	4	fc.Q_rm.4	0.86440671	0.949	0.06670998	0.05260332	0.8688694	0.06623014	0.76675	0.07795886	0.103125	0.14732864	0.046875	0.09375	678.122604	2.90907306	0.03425999	6
24	P	4	fc.P_rm.4	0.86822215	0.957	0.05788474	0.04140838	0.87041099	0.05615887	0.7495	0.09198007	0.12585227	0.13237011	0.046875	0.09375	678.318166	3.10463486	0.03106859	4
25	LQ	4	fc.LQ_rm.4	0.86453577	0.944	0.06614175	0.05328222	0.8684602	0.06619492	0.774	0.07880355	0.103125	0.14732864	0.046875	0.09375	686.860047	11.6465158	0.00043399	9
26	LQP	4	fc.LQP_rm.4	0.86691538	0.958	0.06382764	0.05163433	0.87016498	0.0646899	0.7925	0.07635225	0.103125	0.14732864	0.046875	0.09375	683.356618	8.14308668	0.0025017	7
27	H	4	fc.H_rm.4	0.87384448	0.838	0.06167214	0.05216837	0.86912347	0.06304042	0.8465	0.0767789	0.15520833	0.19321162	0.0625	0.125	685.762866	10.5493351	0.00075115	9
28	LQH	4	fc.LQH_rm.4	0.86453577	0.944	0.06515105	0.0533288	0.86829428	0.06624063	0.79025	0.05615084	0.103125	0.14732864	0.046875	0.09375	686.860047	11.6465158	0.00043399	9

Table S4 - Performance evaluation of the contemporary model based on features and regularization parameters.

fc	rm	tune.args	auc.train	cbi.train	auc.diff.avg	auc.diff.sd	auc.val.avg	auc.val.sd	cbi.val.avg	cbi.val.sd	or.10p.avg	or.10p.sd	or.mtp.avg	or.mtp.sd	AICc	delta.AICc	w.AIC	ncoef	
1	L	1	fc.L_rm.1	0.901274073	0.905	0.034554189	0.022951794	0.885496292	0.032168087	0.81825	0.094982016	0.170833333	0.173405434	0.025	0.05	636.6070354	5.419417881	0.014558862	8
2	Q	1	fc.Q_rm.1	0.904326802	0.901	0.031614058	0.024961538	0.889724798	0.030506851	0.79925	0.085418089	0.145833333	0.175	0.075	0.15	631.6837235	0.496105969	0.170691158	7
3	P	1	fc.P_rm.1	0.896707594	0.927	0.031996577	0.027585614	0.883128652	0.032725845	0.778	0.082595399	0.1625	0.158917053	0.025	0.05	632.3092606	1.121643139	0.124846782	5
4	LQ	1	fc.LQ_rm.1	0.906454461	0.937	0.030396965	0.026198871	0.889640374	0.030748136	0.80675	0.088477398	0.1625	0.158917053	0.075	0.15	641.7741769	10.58655943	0.001099251	11
5	LQP	1	fc.LQP_rm.1	0.90317467	0.926	0.029714702	0.027551268	0.885849144	0.030903027	0.79775	0.12241834	0.1625	0.158917053	0.075	0.15	641.3033479	10.11573036	0.001391029	10
6	H	1	fc.H_rm.1	0.919237238	0.906	0.040501859	0.042932022	0.882008641	0.034271311	0.581	0.300232132	0.158333333	0.125830574	0.075	0.15	666.4036246	35.21600708	4.93E-09	20
7	LQH	1	fc.LQH_rm.1	0.917202086	0.935	0.034450223	0.039456703	0.886490481	0.030762865	0.7625	0.132477671	0.133333333	0.124721913	0.075	0.15	675.5301002	44.34248271	5.14E-11	21
8	L	2	fc.L_rm.2	0.898095198	0.931	0.034656508	0.024186402	0.88415316	0.032607612	0.787	0.143017481	0.170833333	0.173405434	0.025	0.05	636.3961142	5.20849672	0.016178132	7
9	Q	2	fc.Q_rm.2	0.902089816	0.967	0.031228378	0.02304862	0.887921591	0.030256179	0.7965	0.107171203	0.120833333	0.127202813	0.075	0.15	631.1876175	0	0.21874547	6
10	P	2	fc.P_rm.2	0.895681608	0.946	0.029803636	0.031267957	0.882466749	0.034602383	0.805	0.087662231	0.141666667	0.177169097	0.025	0.05	634.8942625	3.706645003	0.03428083	5
11	LQ	2	fc.LQ_rm.2	0.902897149	0.952	0.031587576	0.024889176	0.887816231	0.031608624	0.77675	0.106543809	0.145833333	0.175	0.075	0.15	635.5005176	4.31290013	0.025316561	8
12	LQP	2	fc.LQP_rm.2	0.898910941	0.902	0.031411162	0.028775557	0.884348001	0.034211815	0.80575	0.06575903	0.120833333	0.127202813	0.05	0.1	639.6841365	8.496518957	0.003125672	8
13	H	2	fc.H_rm.2	0.905335968	0.872	0.030481921	0.039551224	0.880788425	0.0335644	0.67025	0.081549065	0.154166667	0.099419612	0.05	0.1	650.2926958	19.1050783	1.55E-05	14
14	LQH	2	fc.LQH_rm.2	0.908842822	0.936	0.028437656	0.028313841	0.888298474	0.02722175	0.8115	0.079466974	0.154166667	0.099419612	0.075	0.15	643.7784802	12.5908627	0.000403523	13
15	L	3	fc.L_rm.3	0.896253469	0.939	0.03370575	0.024903956	0.884318871	0.033257218	0.81275	0.111876047	0.170833333	0.173405434	0.025	0.05	632.5616422	1.374024719	0.110045779	5
16	Q	3	fc.Q_rm.3	0.901038601	0.971	0.030587481	0.02254008	0.88694921	0.029734621	0.78275	0.075110474	0.145833333	0.131497782	0.05	0.1	632.8798102	1.692192711	0.093860765	6
17	P	3	fc.P_rm.3	0.893823059	0.946	0.028604543	0.033187275	0.880486498	0.034796572	0.766	0.19686205	0.141666667	0.177169097	0.05	0.1	638.5187222	7.331104685	0.005597704	5
18	LQ	3	fc.LQ_rm.3	0.901820705	0.952	0.030809882	0.024863074	0.886760876	0.03102871	0.77475	0.073938601	0.170833333	0.173405434	0.05	0.1	637.2318137	6.044196235	0.010652671	8
19	LQP	3	fc.LQP_rm.3	0.894773358	0.959	0.028689147	0.02841611	0.885141139	0.03253143	0.83025	0.106952871	0.095833333	0.082073815	0.025	0.05	641.0421274	9.854509867	0.00158511	7
20	H	3	fc.H_rm.3	0.897001934	0.834	0.034764046	0.037307548	0.879899454	0.039626107	0.744	0.086409876	0.120833333	0.127202813	0.025	0.05	646.4812207	15.29360318	0.000104466	11
21	LQH	3	fc.LQH_rm.3	0.901686149	0.93	0.028340652	0.024457505	0.884564708	0.027862013	0.81425	0.075931438	0.1625	0.109184689	0.05	0.1	636.5259813	5.338363834	0.015161008	9
22	L	4	fc.L_rm.4	0.895942309	0.928	0.032374185	0.025430568	0.884786866	0.033120639	0.7965	0.094546285	0.145833333	0.131497782	0.025	0.05	633.1923591	2.004741561	0.080281406	5
23	Q	4	fc.Q_rm.4	0.898456816	0.944	0.030658061	0.023126077	0.88581312	0.030308204	0.7995	0.085827346	0.145833333	0.131497782	0.05	0.1	635.1735513	3.98593377	0.029812922	6
24	P	4	fc.P_rm.4	0.891586074	0.95	0.028202935	0.032769741	0.880128764	0.034202205	0.7865	0.123810339	0.116666667	0.129099445	0.05	0.1	636.8645365	5.676918962	0.012800053	3
25	LQ	4	fc.LQ_rm.4	0.899213691	0.939	0.030357966	0.025507557	0.885555104	0.031524232	0.796	0.087726849	0.145833333	0.131497782	0.05	0.1	639.5829949	8.395377403	0.003287805	8
26	LQP	4	fc.LQP_rm.4	0.893999664	0.948	0.027619452	0.028133904	0.885089455	0.031693551	0.82775	0.132243147	0.120833333	0.127202813	0.025	0.05	635.66988	4.482262544	0.023260986	4
27	H	4	fc.H_rm.4	0.894243546	0.864	0.032506372	0.034950634	0.880632842	0.038860335	0.7205	0.068476273	0.120833333	0.127202813	0.025	0.05	643.8178093	12.63019183	0.000395665	9
28	LQH	4	fc.LQH_rm.4	0.898961399	0.915	0.028454598	0.024248859	0.885189638	0.029546927	0.79525	0.074069224	0.1625	0.109184689	0.05	0.1	640.130175	8.942557465	0.002500849	9

Table S5.- Number of training and test simples, and average and standard deviation of training and test AUC, AUC standard deviation, and percentage of contribution and permutation of each variable for the contemporary and projection models.

	Historical	Contemporary
**N Training - Test**	40-9	38-9
**Training AUC (±SD)**	0.865±0.024	0.9±0.021
**Test AUC (±SD)**	0.842±0.07	0.879±0.057
**AUC Standard Deviation**	0.056	0.042
**% contribution**		
**Bio 01**	0±0	0.004±0.018
**Bio 08**	8.262±8.729	0.755±1.033
**Bio 09**	73.079±13.838	91.69±5.488
**Bio 14**	0.153±0.282	1.377±2.114
**Bio 15**	4.815±11.48	0.061±0.208
**Autumn sd**	3.421±4.608	0.174±0.737
**Spring mean**	0.663±1.161	1.214±2.177
**Spring sd**	8.378±5.285	4.328±4.521
**Summer sd**	1.23±2.262	0.398±0.559
**% permutation (±SD)**		
**Bio 01**	0±0	0.049±0.217
**Bio 08**	24.348±24.072	2.266±3.465
**Bio 09**	48.555±23.289	79.6502±19.45
**Bio 14**	0.136±0.390	6.594±12.373
**Bio 15**	3.92±10.704	0.402±1.242
**Autumn sd**	4.641±8.066	0.163±0.539
**Spring mean**	0.805±1.304	3.367±4.298
**Spring sd**	15.229±13.562	7.04±12.832
**Summer sd**	2.364±4.032	0.4696±0.884

FIGURES

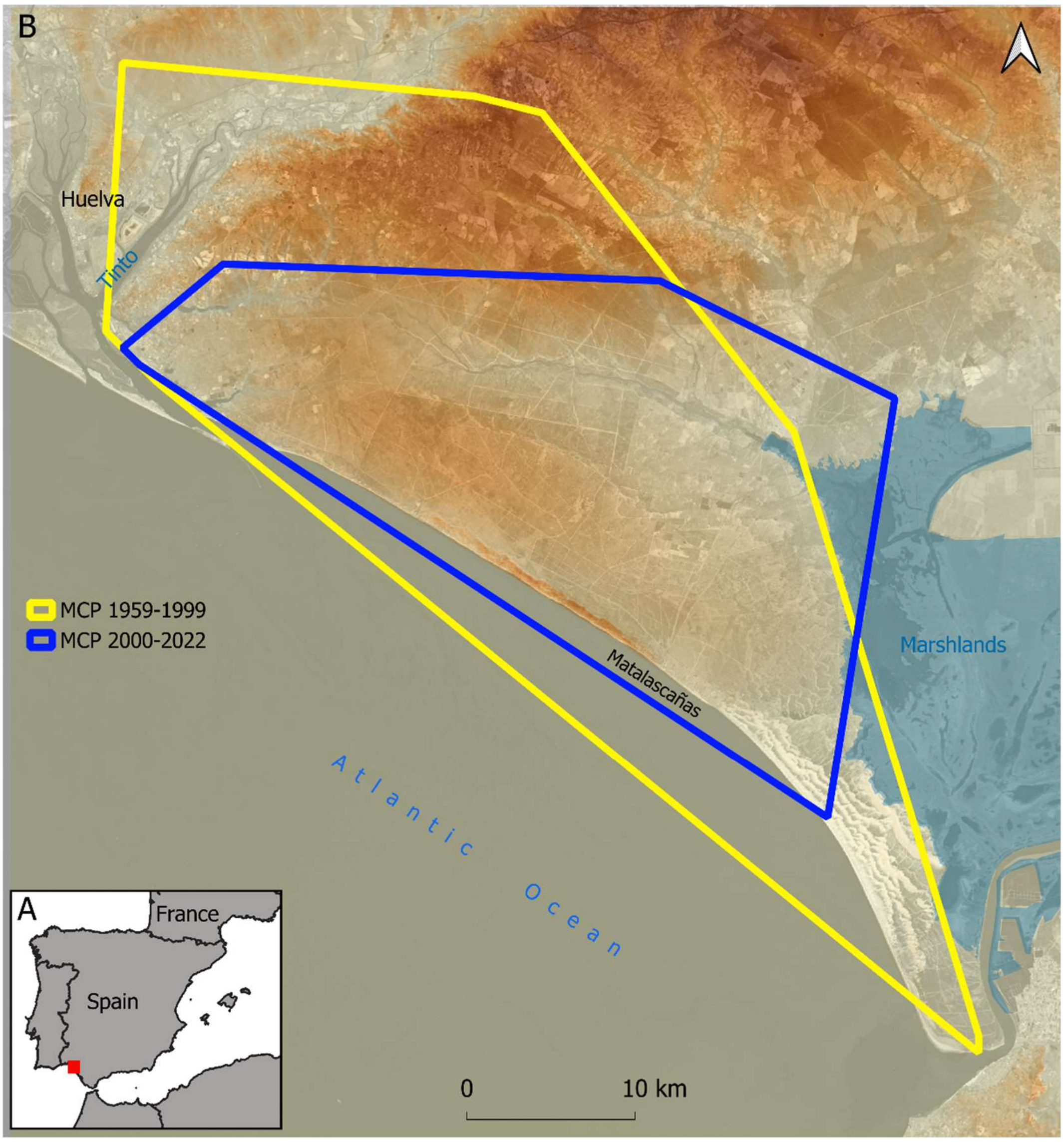

Figure S1- Minimum Convex Polygon (MCP) for the period 1959-1999 (yellow) and MCP for the period 2000-2022.

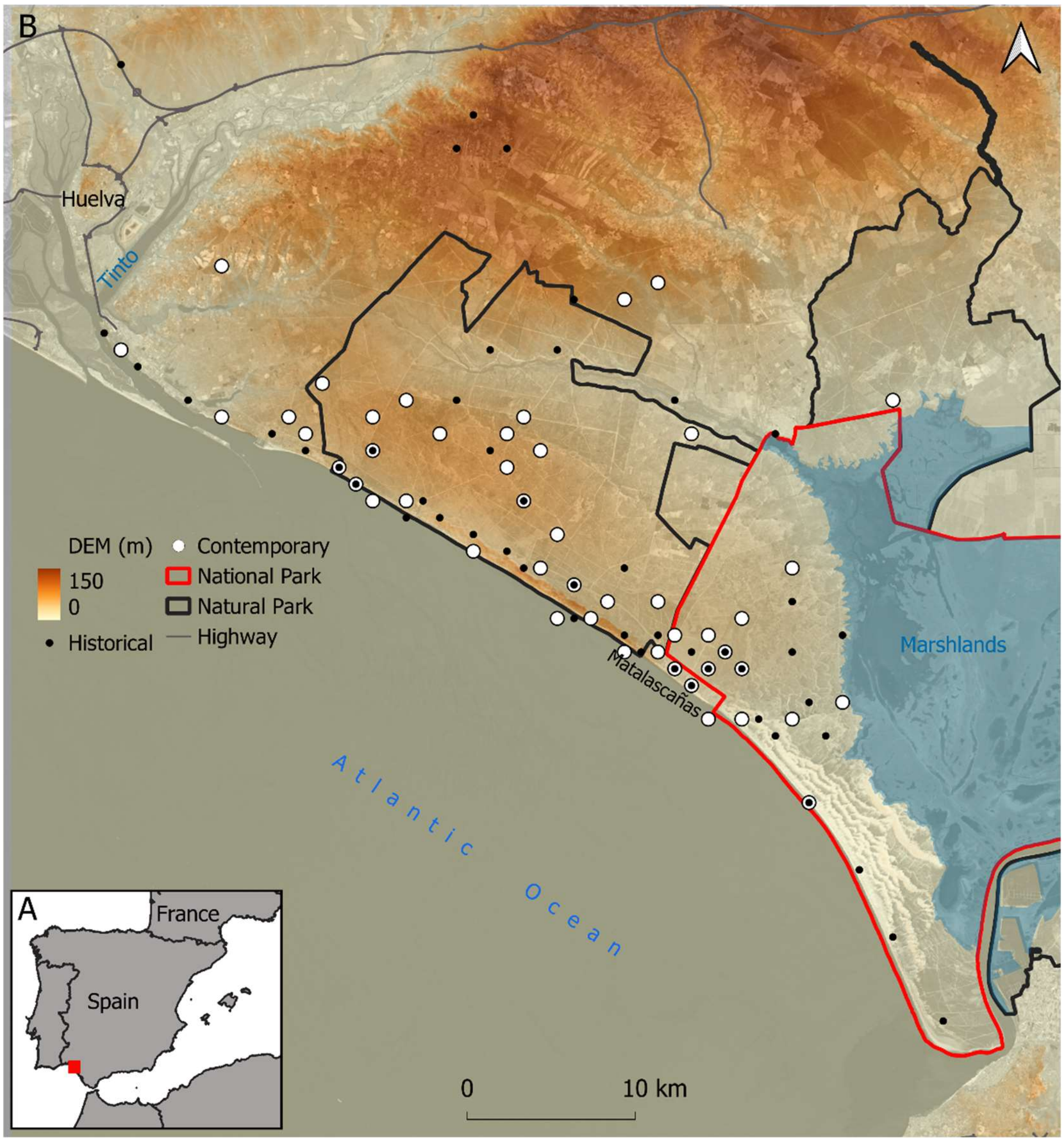

Figure S2-Occurrence data used to run ecological models in historical (white and black dots) and contemporary periods (black dots).

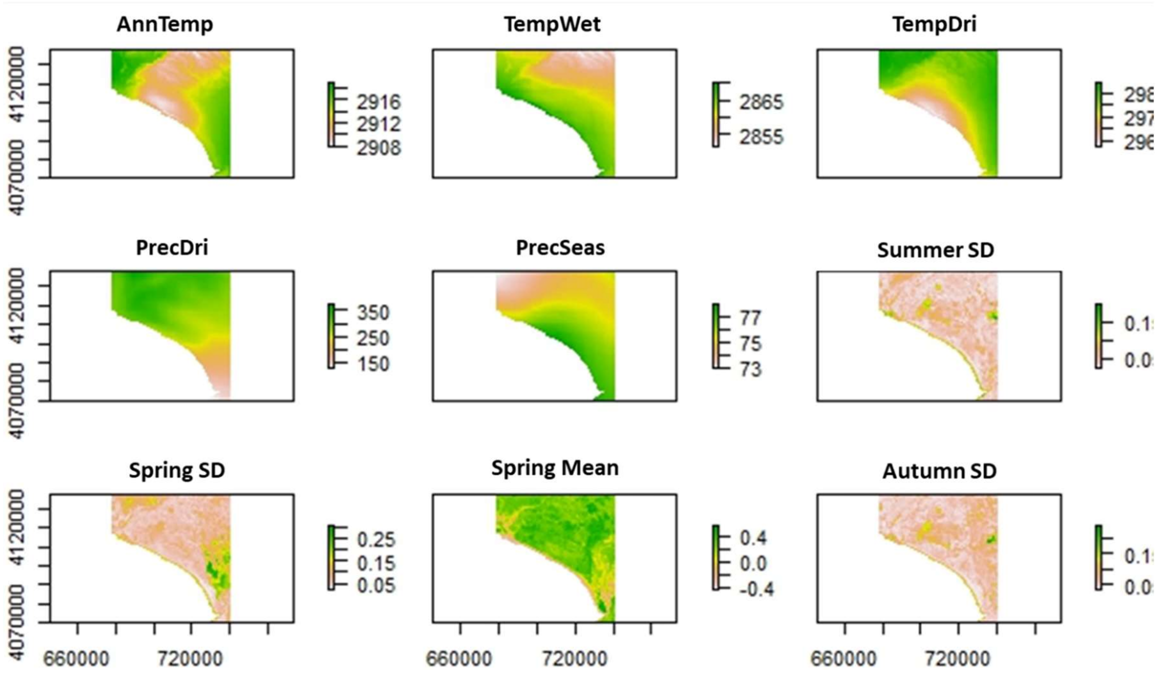

Figure S3- Spatial representation of the variables used in the historical models.

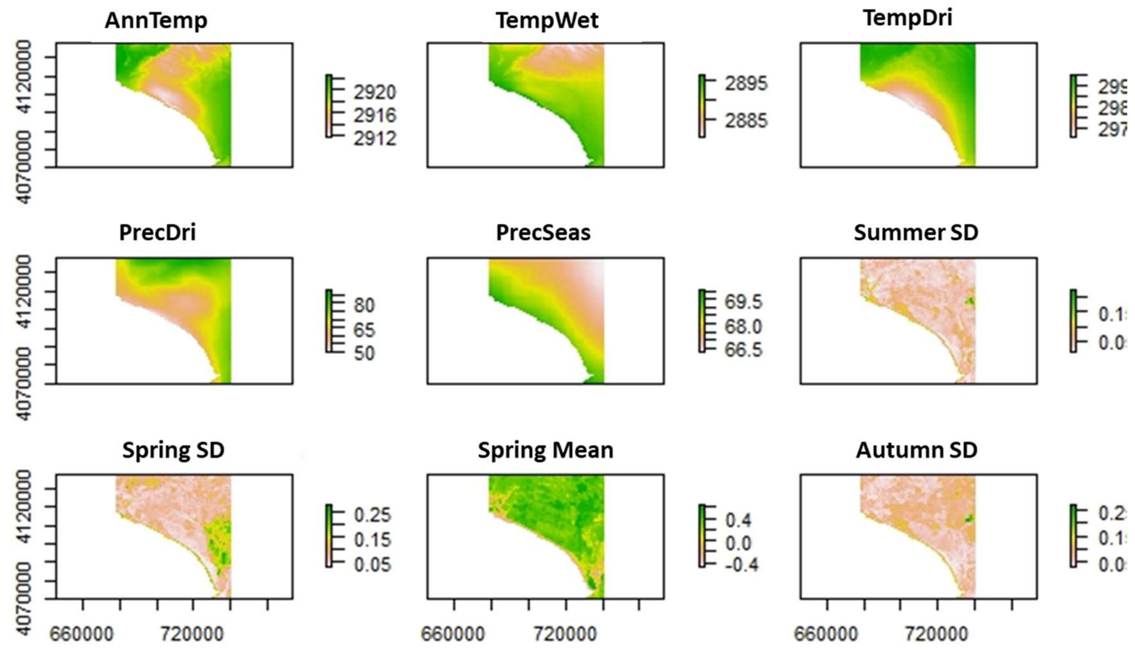

Figure S4- Spatial representation of the variables used in the contemporary models.

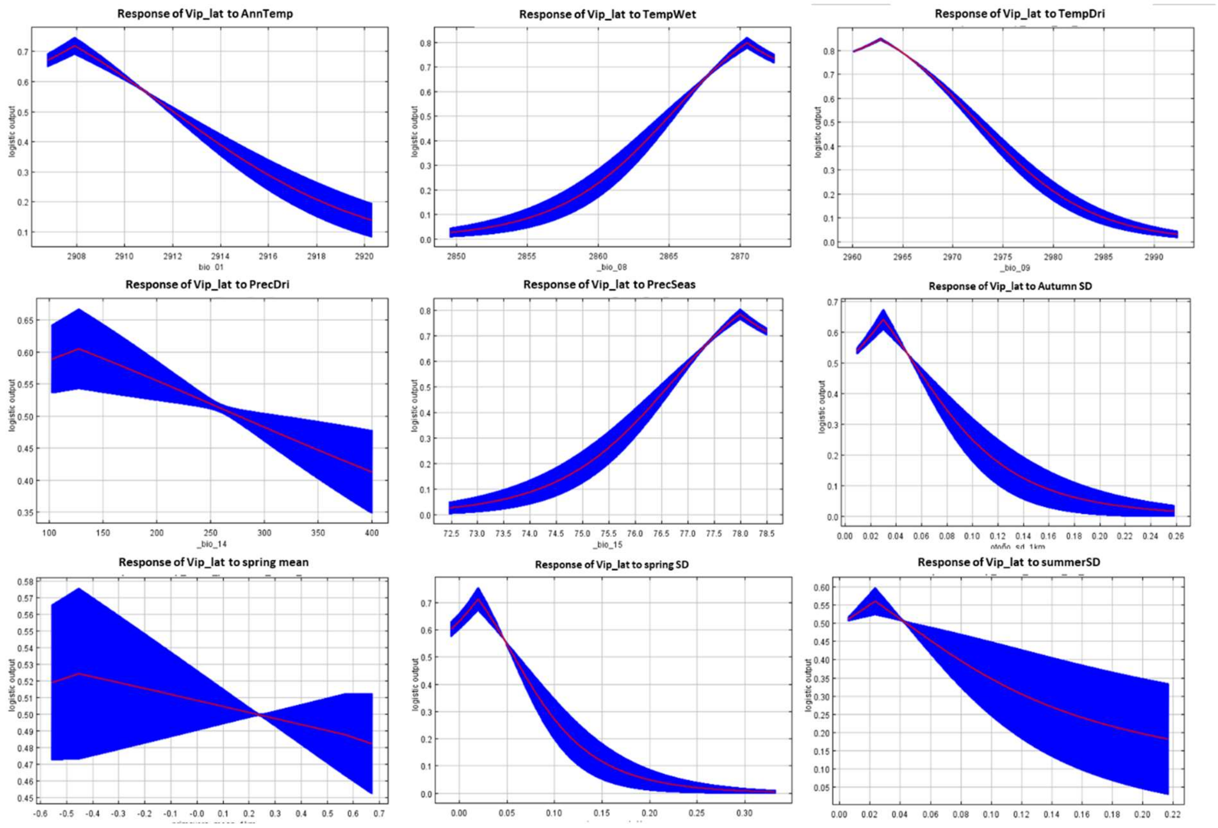

Figure S5- Response curves for the variables considered in the historical model.

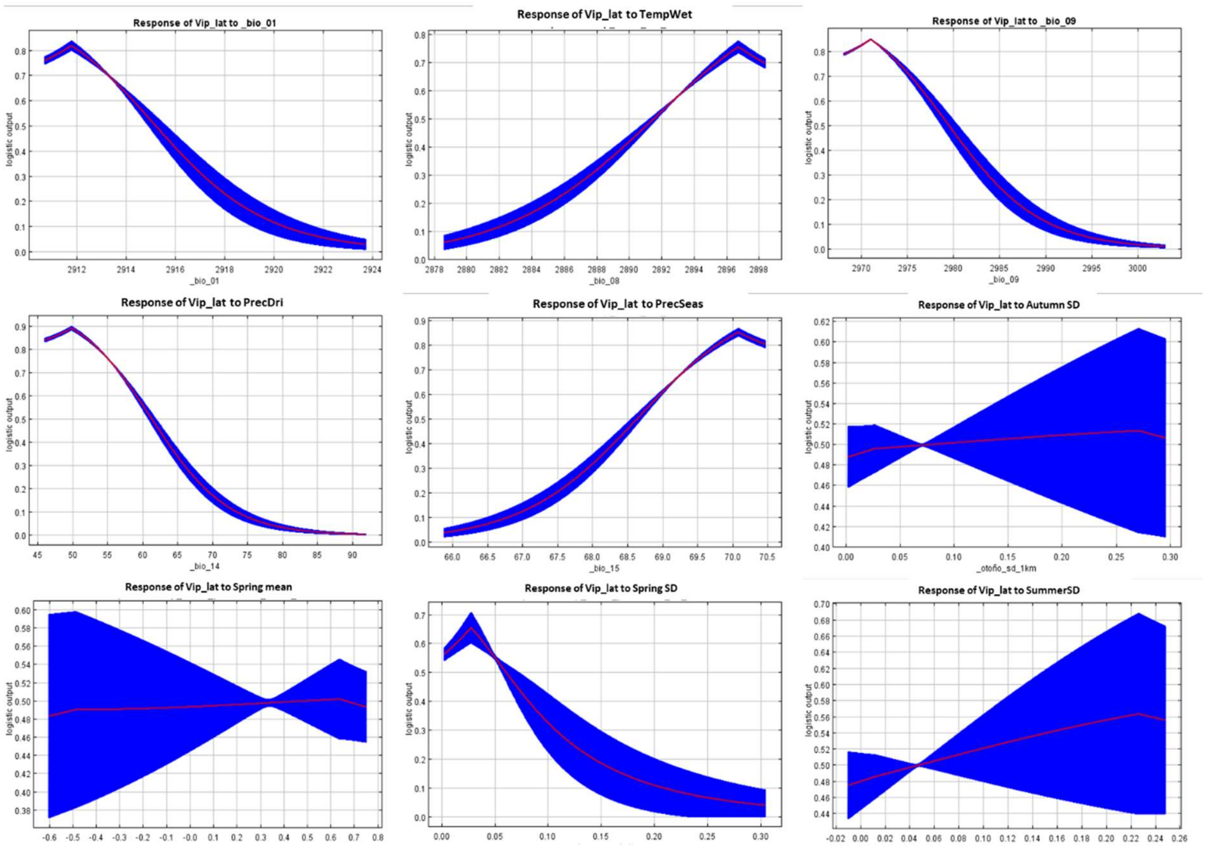

Figure S6- Response curves for the variables considered in the contemporary model.

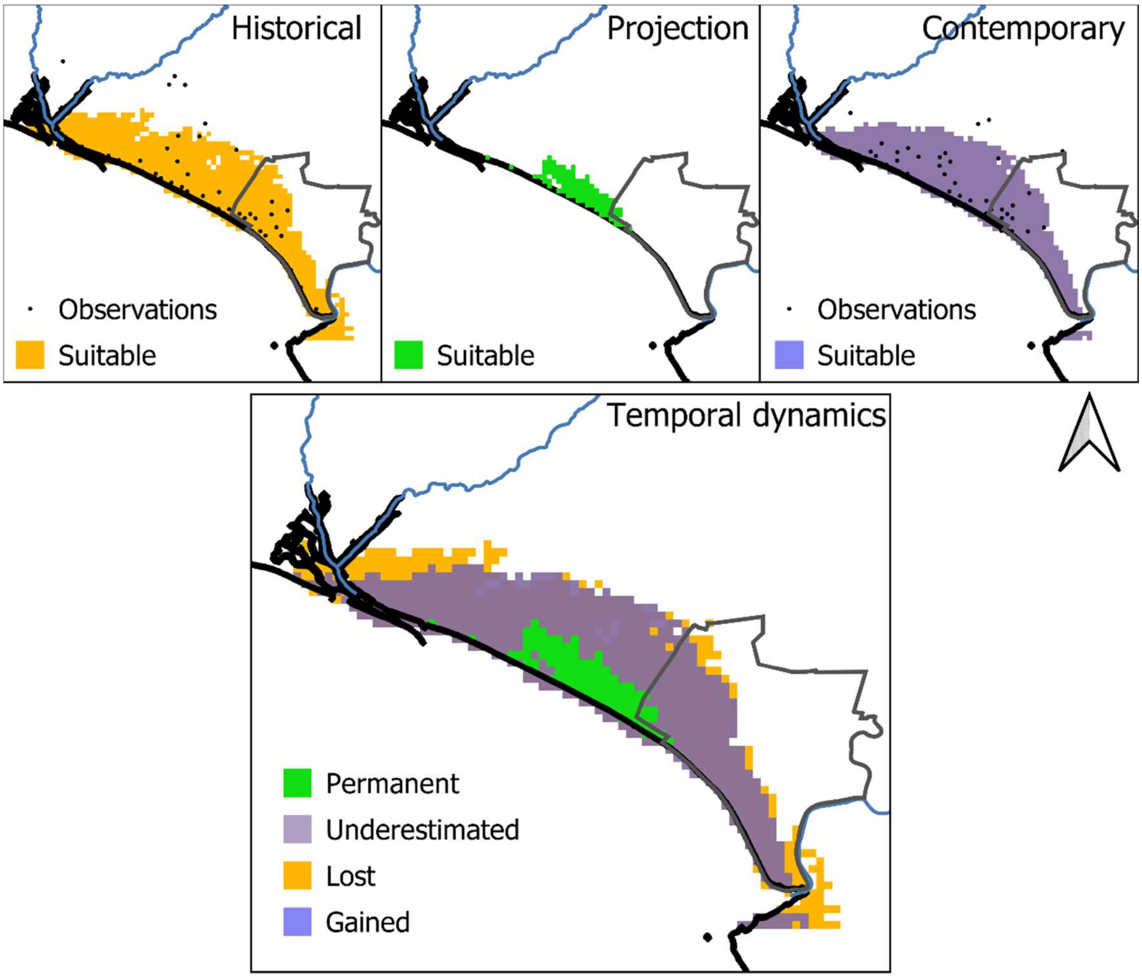

Figure S7- Historical, contemporary and projection of suitable habitats and temporal dynamics of habitat suitability for Lataste's viper in Doñana, considering the 10th percentile logistic threshold. Lost habitats refer to pixels identified in the Historical only; permanent suitable habitats to pixels identified in Historical, Projection and Contemporary; underestimated suitable habitats refer to pixels identified in the Contemporary and Historical; and gained suitable habitats refer to pixels identified in the Contemporary only.
